# Nanophotonic crystals on unpolished sapphire substrates for deep-UV light-emitting diodes

**DOI:** 10.1038/s41598-021-84426-z

**Published:** 2021-03-02

**Authors:** Tinh Binh Tran, Feras AlQatari, Quang-Ho Luc

**Affiliations:** 1 Colleges of Nanoscale Science and Engineering, SUNY Polytechnic Institute, Albany, NY 12203 USA; 2 Advanced Semiconductor Laboratory, KAUST, Thuwal, 23955 Saudi Arabia; 3 Department of Materials Science and Engineering, National Chiao Tung University, 1001 University Road, Hsinchu, 300 Taiwan

**Keywords:** Engineering, Materials science

## Abstract

A new method has been established and employed to create a random nanophotonic crystal (NPhC) structure without photolithography on the unpolished side of a single-side-polished sapphire substrate. This nano structure has potential use in enhancing the light-extraction efficiency (LEE) of deep ultraviolet light-emitting diodes (DUV-LEDs), and has never been built for DUV-LED applications before. Two mask layers in the nano scale (Au and SiO_2_) were used to create the NPhC and observed using scanning electron microscopy to have an average height of 400 nm and various sizes from 10 to 200 nm. Finally, a conventional DUV-LED and a DUV-LED device with NPhC were simulated using 2D Lumerical Finite-Difference Time-Domain (FDTD) for comparison. The results show that the LEE of the DUV-LED device with this NPhC integrated was significantly directly enhanced by up to 46% and 90% for TE and TM modes, respectively, compared to the conventional DUV-LED device. Thus, this NPhC is believed to be a new, key technique to enhance the LEE of DUV-LEDs.

## Introduction

Single crystal sapphire (Al_2_O_3_) wafer has become one of the most popular substrates for growing III–V semiconductor materials for optoelectronic and power electronic devices since its initial development in the 1960s^1^. Sapphire substrate in particular is the most widely used in III-Nitride semiconductor materials for light-emitting diodes (LEDs)^2^, deep ultraviolet LEDs (DUV-LEDs)^3–6^, laser diodes^7^ and high electron mobility transistors^8,9^ compared to other substrates such as SiC^10,11^ and Si^12–15^, due to the low lattice mismatch, large diameter, high optical transparency, high thermal stability and corrosion resistance, low cost, etc^16–19^. However, sapphire substrates pose many other problems such as cutting and polishing after chip growth and fabrication due to the high hardness and its orientation. For DUV-LEDs that emit from the back side, internal reflection from the unpolished SSP sapphire surface reduces the light-extraction efficiency (LEE) significantly, in addition to losses due to low reflectivity on the polished top side of the device. Recently, many techniques have been advanced to increase the LEE such as using a super-lattice hole-spreading layer with an Al reflector^20^; flip chip DUV-LEDs^21^ with a roughened AlN template surface^22^; highly reflective photonic crystals on a p-AlGaN contact layer on top of the DUV-LED structure^23^; and highly reflective layers and a patterned sapphire substrate^24^. However, improving the LEE by making a nanophotonic crystal (NPhC) on the unpolished side of the single-side-polished (SSP) sapphire substrate has not been done before (the polished side sapphire substrate is for semiconductor growth and the unpolished side is for creating NPhC, where the UV light transmits to the air). In this work, we will introduce an etching technique that needs neither a photoresist nor photolithography to etch and create nano features on a c-sapphire substrate by using inductively coupled plasmas (ICP) to create a NPhC with various sizes down to 10 nm. This method is a simple and easy way to obtain NPhC on the unpolished side of the SSP sapphire substrate, as compared to other methods such as nanoimprint lithography^25,26^ that are limited to create patterns on the front (polished) side and in a limited area. The etching process used in this work combined Boron trichloride (BCl_3_), Hydrogen bromide (HBr), and Argon (Ar) gases with two nanomask layers. The nanomask formation processes and the resulting etch profile were observed by scanning electron microscopy (SEM). To prove that this NPhC can be used to enhance the LEE of DUV-LEDs, a 2D Lumerical Finite-Difference Time-Domain (FDTD) simulation was used to model a DUV-LED device with this NPhC as well as a conventional device for comparison.

## Materials and methods

**Figure 1:**
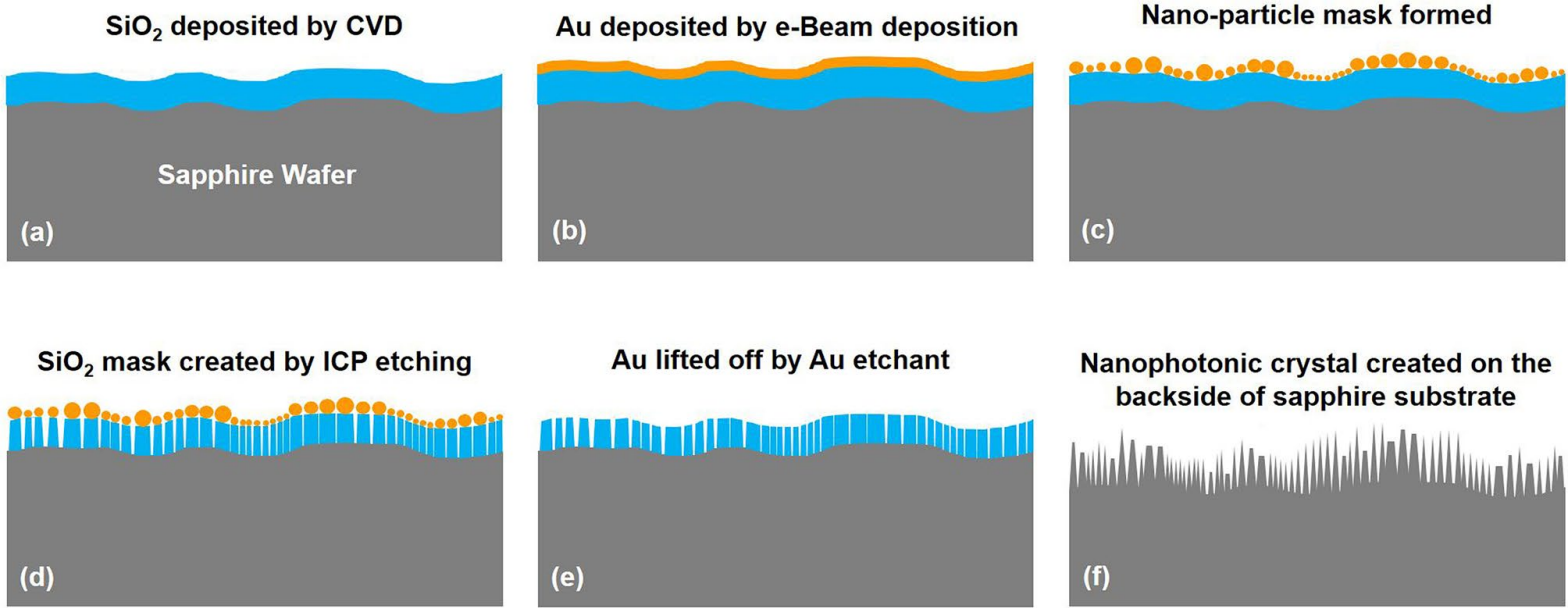
Fabrication process of the NPhC on the unpolished side of SSP sapphire substrate. (**a**) A SiO_2_ is deposited on the unpolished side, (**b**) a thin Au layer is deposited over the SiO_2_, (**c**) the first Au nanomask forms under high temperature, (**d**) the Au nanomask was employed to etch the SiO_2_ nanomask, (**e**) Au nanomask removed, (**f**) sapphire etched through the SiO_2_ nanomask, and SiO_2_ removed to leave the final NPhC sapphire substrate.

An SSP 2-inch c-sapphire substrate was first cleaned by treatment for 60 s in each of acetone and then isopropyl alcohol, then for 180 s in de-ionized water. After, a 300 nm-thick layer of SiO_2_ was vapor-deposited on the unpolished side of the substrate (Fig. 1a). The substrate was then loaded into an e-Beam evaporator system to deposit a 10 nm-thick Au layer on the top (Fig. 1b). The deposition rates are approximately 1.1 and 1.0 nm/sec for SiO_2_ and Au, respectively. Next, the sapphire substrate was placed in a rapid thermal annealing (RTA) furnace at 700 °C for 5 min to form the first multi-Au nanomask (nanoparticle-like)^27^ as shown in Fig. 1c. After that, the SiO_2_ was etched to create the second nanomask (Fig. 1d) using reactive-ion etching (RIE). The residual Au nanomask was then removed using gold etchant, as shown in Fig. 1e. Finally, the sapphire substrate etching process was carried out in an ICP etching system (Oxford Plasma Lab System 100) using BCl_3_, HBr and Ar gases, and the SiO_2_ was removed using HF to leave the NPhC sapphire substrate (Fig. 1f).

## Results and discussion

**Figure 2:**
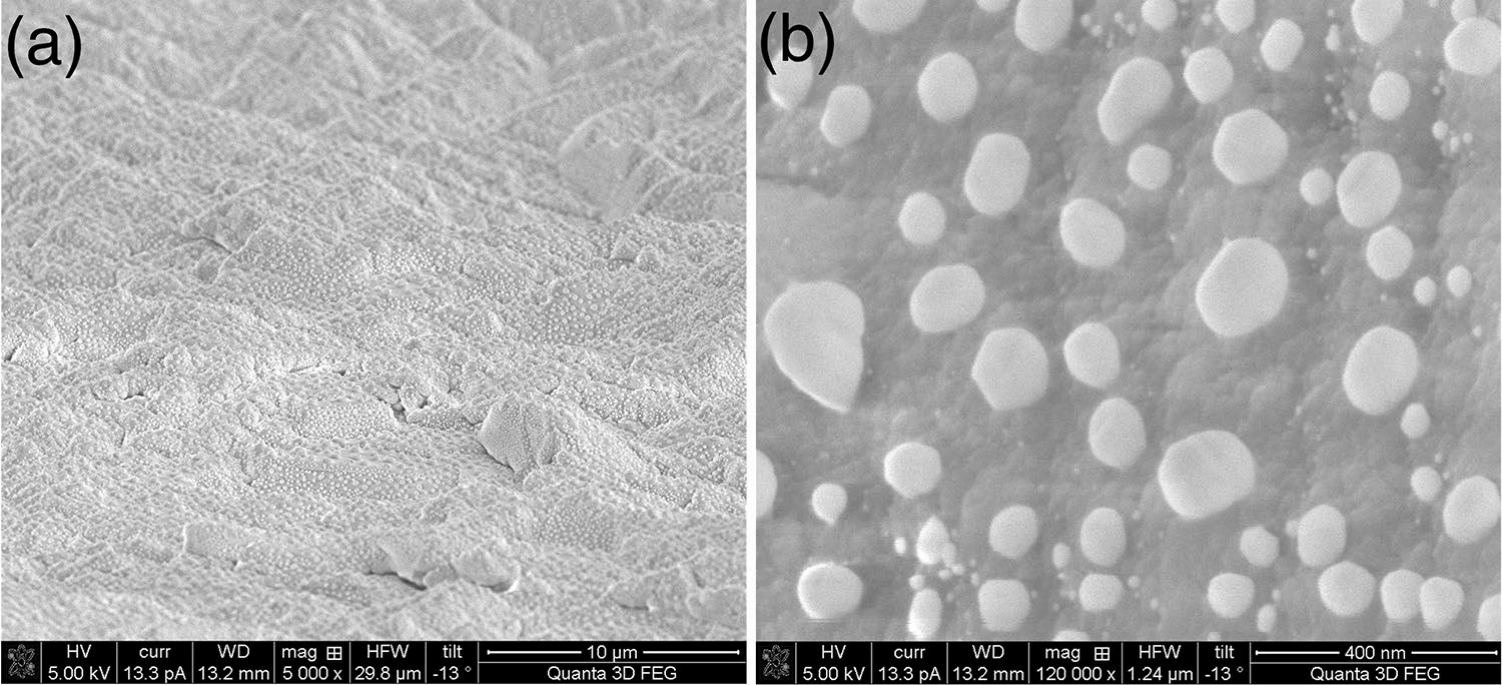
(**a**) SEM image showing the Au nanomask on a large, unpolished area of sapphire substrate; (**b**) a close-up showing the distribution in size and position of Au nanomask particles.

The etched sapphire substrate shown in this article was produced using optimal conditions including steps such as annealing and etching through both nanomasks. Figure 2a shows the unpolished side of the SSP sapphire substrate after a thin Au layer was annealed at a high temperature of 700 °C and the Au nanomask that formed under long exposure to that temperature. The Au nanomask particles range in sizes from less than 10 nm to around 200 nm and are mostly convex but irregular, as shown in Fig. 2b. The different sizes obtained may be due to the high roughness (0.8–1.2 µm) of the unpolished substrate surface, leading to variations in forces and in gold film thickness. We found that the larger Au nanoparticles tend to be located on the top of the hills while the smaller ones lie mostly on the hillsides or in valleys. This mask-making technique generates a random pattern, and the nanomask size is difficult to control because of the unpolished surface.

**Figure 3:**
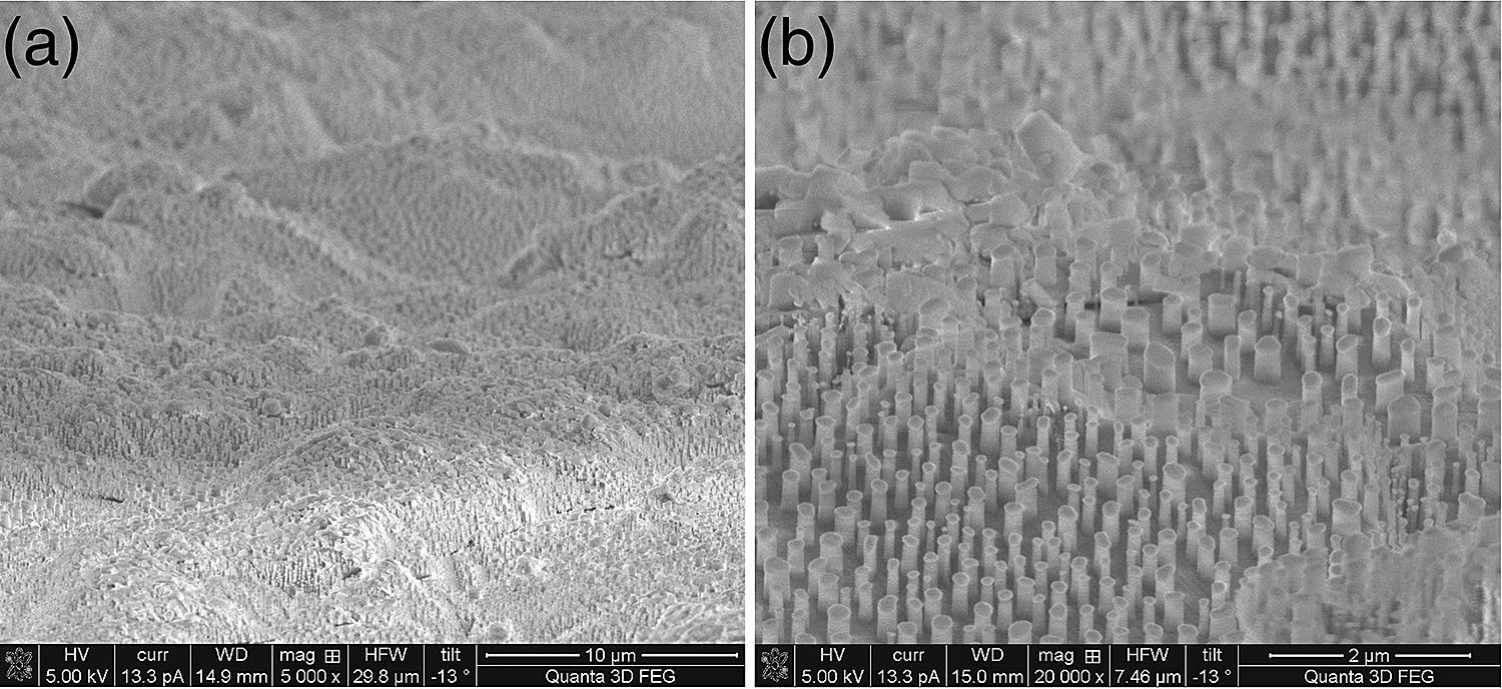
(**a**) The SiO_2_ layer immediately after RIE plasma etching, with Au nanomask still in place; (**b**) a close up to show verticality of SiO_2_ nanopillars.

In order to get a perfectly anisotropic etching result for the SiO_2_ nano mask, RIE plasma etching was carried out with the RF power at 100 W and chamber pressure at 10 Torr. The gas flows were set at 40 and 5 sccm for C_4_F_8_ and O_2_, respectively. The etching rate was estimated at 4 nm/s. Figure 3a shows the perfectly vertical nanopillars achieved on the SiO_2_ surface (as the second nanomask) with the Au nanomask particles still on the top of each SiO_2_ pillar (Fig. 3b).

**Figure 4:**
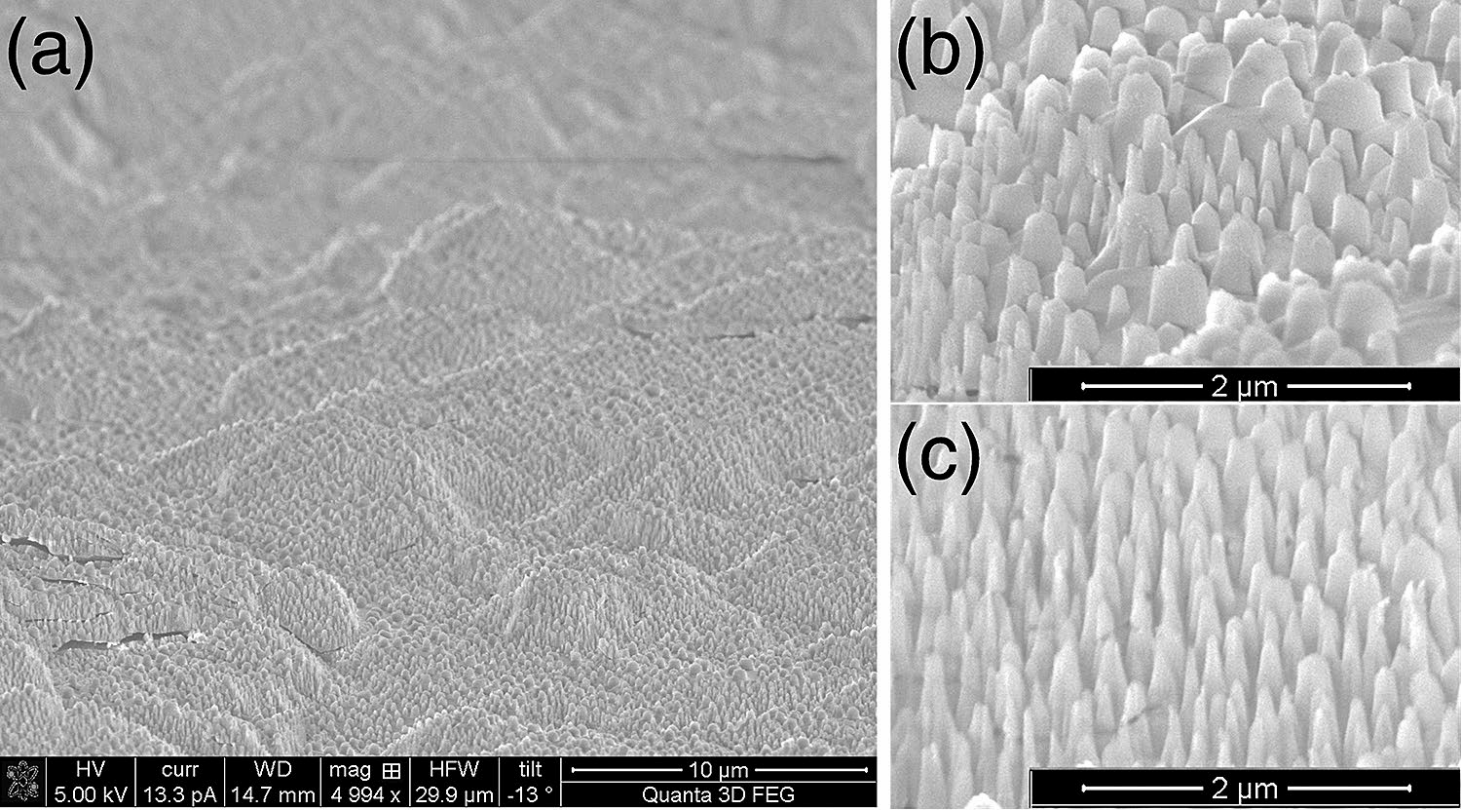
NPhC created on the unpolished side of the SSP sapphire substrate (**a**). Subfigure (**b**) shows larger NPhC which tend to form on hilltops; (**c**) the smaller NPhC form mostly on hillsides.

To prevent contamination, gold etchant was used to remove the Au nanomask. The sapphire substrate was then loaded into an ICP system to etch the sapphire substrate. During etching, the chamber pressure was kept at 4 mTorr together with the bias power of 100 W, with a constant flow of the BCl_3_ and HBr gases at a 4:1 ratio and maintained Ar at 3 sccm. The etching rate is estimated to be about 2 nm/s which is considered the best rate for forming the NPhC structure shown in Fig. 4. The structure comprises spikes of varying dimensions. Diameters were distributed with modes at 50, 100, 200, 300 and 400 nm; height was typically 400 nm; inter-spike spacing averaged 200 nm.

**Figure 5:**
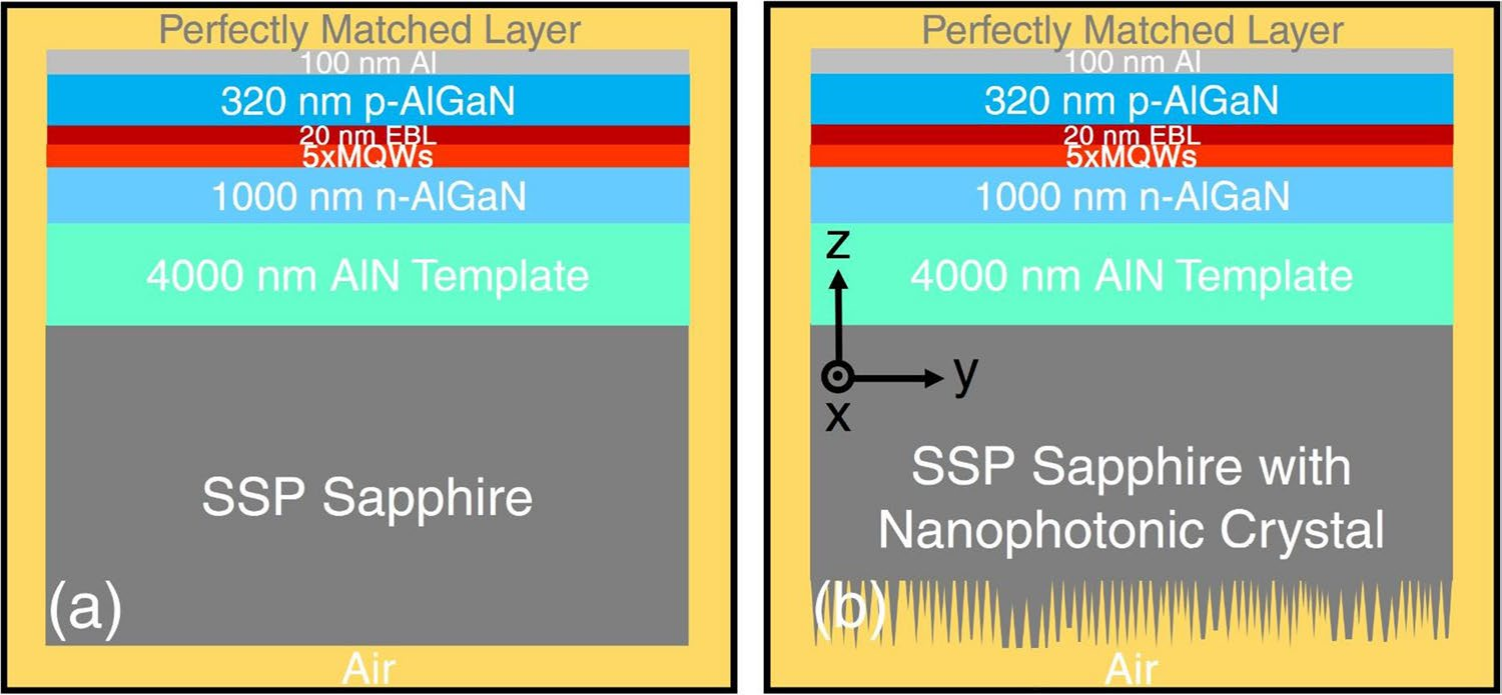
Schematic illustration of the simulated model DUV-LED devices with NPhC (**b**) while the model for the conventional DUV-LED device is almost the same except the sapphire substrate without NPhC (**a**).

Because this is the first time such a NPhC structure has been created on a SSP sapphire substrate, we simulated its performance in a DUV-LED using the 2D FDTD method, comparing DUV-LEDs with and without the NPhC structure. The FDTD simulation was built to investigate the effect of the NPhC on LEE in particular, for both transverse electric (TE) and transverse magnetic (TM) polarization modes. Figure 5 shows two DUV-LED devices with 100 nm-thick Al reflectors on each p-type layer. The conventional DUV-LED device (Fig. 5a) differs from the device with NPhC only by not having the etched NPhC surface on the unpolished side of sapphire substrate (Fig. 5b). In the simulation model, we used a flat surface and integrated five different NPhC sizes periodically, as mentioned above. We could not make a rough surface to approximate as-received SSP sapphire substrate due to the limitation of the software. However, with a small spacing between each NPhC and high NPhC density, this should have no effect on the simulation result. A single Gaussian-shaped dipole source is applied at the middle of the multiple quantum wells (MQWs) in each structure. The emission and full-width at half-maximum wavelengths of the dipole source are set at 280 and 10 nm, respectively, and polarized in the parallel and perpendicular directions to the MQW plane to excite the TE and TM modes, respectively. The refractive indices of AlN, AlGaN and sapphire are 2.0, 2.6 and 1.8, respectively; these have a big impact on the LEE of DUV-LEDs^28^. LEE is basically defined as the ratio of emission power collected by monitors surrounding the device and main emission surface directions to the total emission power of the DUV-LED, and is determined by the Poynting vectors along each emission direction and the total Poynting vector^29^. Therefore, the Poynting vectors are used to determine the LEE of a DUV-LED device, calculated below the surface of the perfectly matched layers. The emission light from the back side of the DUV-LED device and from other sides will be collected for both DUV-LED devices with and without NPhC, for which the monitors were built around the device (black square line) at the edge of the yellow background in Fig. 5 with distance of 300 nm away from the structure.

**Figure 6:**
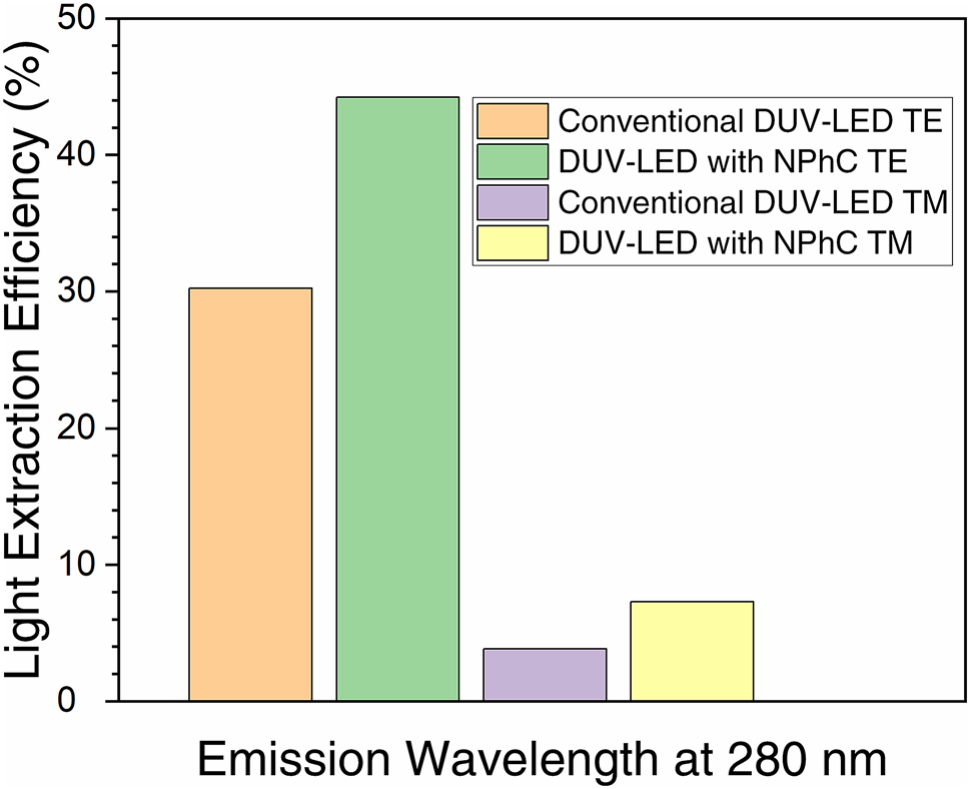
Simulation results: NPhC etching enhanced the LEE up to 46 and 90% for TE and TM modes, respectively, relative to the conventional DUV-LED device.

Figure 6 shows the LEE result of the two DUV-LED devices, with and without NPhC, in both TE and TM modes at the emission wavelength of 280 nm. The simulation reveals a markedly higher LEE for the DUV-LED device with NPhC over the conventional structure. In the TE mode, LEE was increased by 46% (from 30 to 44%), and by 90% (3.8 to 7.3%) for the TM mode. This NPhC structure hence shows great promise for use in DUV-LED applications.

**Figure 7:**
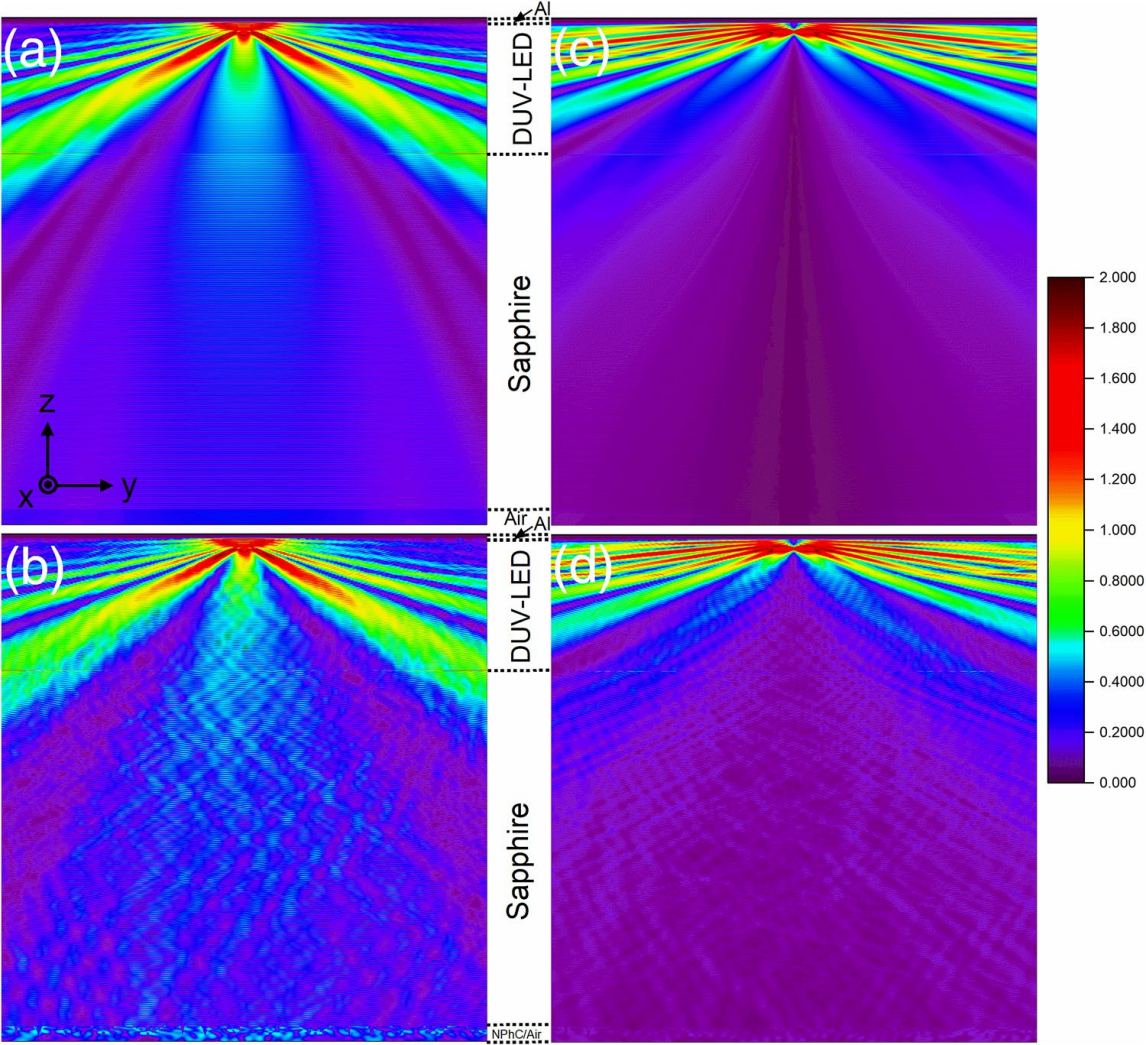
The cross-sectional view of the EFI for TE (**a**, **b**) and TM (**c**, **d**) modes for the conventional DUV-LED (**a**, **c**) and the DUV-LED device with NPhC structure (**b**, **d**), respectively. The NPhC, sapphire, DUV-LED, and Al layers are indicated. The color bar on the right shows the normalized EFI.

Figure 7 shows the cross-sectional electric field intensity (EFI) distribution for the TE and TM modes of the two DUV-LED devices. In the TE mode, the light can be emitted in the *y* and *z* directions since the dipole source is polarized in the *x*-axis. Figure 7b clearly shows the effect of the NPhC on the light distribution inside and outside the device: we can easily see the oscillation of the light inside the sapphire substrate and a significant enhancement to the emitted light at the interface of the NPhC structure and the air which could not be found in the DUV-LED without NPhC. The DUV-LED device with NPhC shows high uniformity in light distribution and brighter outside the device. Especially at the interface of the NPhC and air, light extraction can be seen clearly, as compared to the conventional DUV-LED does in the Fig. 7a. Thus, the NPhC has directly enhanced the light extraction efficiency in the TE mode much more than that of the conventional DUV-LED. For the TM mode, the dipole light source is polarized to the z-axis, so that the light is emitted mostly to the lateral directions as shown in Fig. 7c and d. In this case, too little light escaped at the bottom of the DUV-LED device to see strong oscillations as in the case of the TE mode. However, the effect of the NPhC inside the structure can also be seen from middle of device down to the NPhC-air interface. Overall, the NPhC significantly enhanced the LEE of DUV-LED device which can be easily trusted from the EFI result.

## Conclusion

We have successfully established the most convenient technique, to date, for fabricating NPhC structures on a SSP sapphire substrate using neither photoresist nor photolithography. Unlike conventional photolithography, our technique can be applied to non-flat surfaces such as unpolished SSP sapphire substrates. A NPhC with features as small as 10 nm in diameter can be obtained by using this technique. Small features like this normally require complicated and costly techniques such as e-Beam lithography and nanoimprint lithography where the working area is usually limited. A NPhC structure of this type was integrated into a DUV-LED structure and compared with a conventional DUV-LED in the Lumerical FDTD simulation. The simulation result shows very impressive LEE improvements of 46% (TE) and 90% (TM) over the conventional DUV-LED structure, as well as compared to other techniques^30,31^. LEE enhancement should depend on the size, height, and also the spacing between NPhCs but more studies needed to understand this dependence. The nanophotonic crystal created using this technique promises to be useful in future high-LEE and high external quantum efficiency DUV-LED devices.

## Data Availability

Research data are not shared.
